# Other-regarding attention focus modulates third-party altruistic choice: An fMRI study

**DOI:** 10.1038/srep43024

**Published:** 2017-02-21

**Authors:** Bastian David, Yang Hu, Frank Krüger, Bernd Weber

**Affiliations:** 1 Center for Economics and Neuroscience, University of Bonn, 53127, Germany; 2 Department of Epileptology, University Hospital Bonn, 53127, Germany; 3 Department of Psychology, George Mason University, VA 22030, USA

## Abstract

Third-party altruistic decision-making has been shown to be modulated by other-regarding attention (e.g., focusing on the offender’s crime or the victim’s situation especially in judicial judgment). However, the neural mechanisms underlying this modulation remain poorly understood. In this fMRI study, participants voluntarily decided if they wanted to punish the first-party offender or help the second-party victim using their own monetary endowment in an unfair context. Particularly, before deciding they were asked to focus on the (un)fairness of the offender proposing the offer (offender-focused block, OB), the feeling of the victim receiving this offer (victim-focused block, VB), or without any specific focus (baseline block, BB). We found that compared to BB participants punished more frequently and prolonged help choices in OB, whereas they helped more frequently in VB. These findings were accompanied by an increased activation in the temporo-parietal junction (TPJ) during decision making in OB and VB. Moreover, regions relevant to cognitive control (esp. IFG/AI and the dorsal anterior cingulate cortex) were strongly recruited during specific choices conflicting the attention focus (e.g., choosing help in OB). Our findings revealed how other-regarding attention modulates third-party altruistic decision-making at the neural level.

Social norms refer to the standard of behaviors based on common beliefs accepted by groups or societies about how people should behave in a given situation, such as justice, fairness or cooperation^1^. These normative behaviors play a crucial role in developing and maintaining the unique human society^2^. The violation of social norms (e.g., inequity, injustice) is omnipresent in our world, rendering the consequential enforcement of norms as indispensable. Despite not being directly affected by such violations, third-party observers usually take actions to punish norm violators even by sacrificing their own interests (i.e., costly punishment), representing the key approach to enforce social norms^2^,^3^. One of the most important examples that apply third-party enforcement mechanism to our life is the judiciary system, in which judges can sentence to punish law violators, given the intention and severity of the committed crime, as well as compensate the affected victim.

Fehr and Fischbacher provided first empirical evidence of third-party punishment adopting an economic paradigm^2^. They found that third-party decision-makers punished offenders, who violated either fairness (e.g., by unfair allocations in a dictator game) or cooperation norms (e.g., defecting their partners in a prisoner’s dilemma) to maximize their self-interest. Here, the degree of third-party punishment by sacrificing selfish interests increased with the severity of the norm (fairness) violation. Similar findings have been replicated in later studies^4^,^5^. As third-parties’ economic payoffs were not affected by the violators’ unfair actions, the only motive for choosing to punish is to enforce the social norm and to deter others from wrongdoing, which in turn benefits members of the whole society in the long term, and can therefore be regarded as an altruistic behavior^6^.

However, punishment is not the only conceivable altruistic response in such contexts. It has been shown that third-party observers also helped/compensated unknown victims (i.e., second-parties) using their own monetary endowment in similar unfair situations, if both help and punishment choices were provided^7^,^8^. This finding suggests that people try to upkeep social norms like justice or fairness via different approaches driven by different other-regarding concerns. In general, two major categories of underlying norm concerns exist: retributive or compensatory concern to either punish the offender or to help the victim, respectively^9^,^10^,^11^.

A potential explanation for the diversity of altruistic choices is that the final altruistic decision might be driven by the corresponding concern that is more strongly attended and thereby more salient at the moment of the decision. If the proposed explanation holds true, our opinions and decisions might be greatly shaped by the focus of information provided from different explicit sources such as media^9^,^10^. Consequently, third-party altruistic choices could be influenced by manipulating the attention focus on different contextual aspects.

To our knowledge, very little is known about this crucial issue. In a recent behavioral study^12^, participants read a series of crime descriptions and were asked to choose between different sanctions implementing different concerns to achieve justice. Afterwards, some of them were instructed to focus on the offender-relevant information (e.g., the offender’s intentions and goals; offender-focused condition), whereas others were asked to consider the victim (e.g., how they were affected by the crime; victim-focused condition). After that, participants were asked to choose again among the same sanctions. As expected, people in the victim-focused condition decreased the frequency to choose sanctions directed at punishing the offender (i.e., retributive concern), but increased the frequency to choose sanctions in favor of restoring the victim (i.e., compensatory concern). People in the offender-focused condition did not change their behavior, given that the default choice in this task was the retributive sanctioning. Importantly, this study employed hypothetical crime descriptions as stimuli so that participants knew their decision would not be implemented, which has been shown not to reflect real life decisions, especially in the moral domain^13^.

A key ability of social cognition which serves altruistic decisions is the capacity to understand others’ mental (affective) states, beliefs, and intentions, often referred to as theory-of-mind (ToM)^14^ or mentalizing^15^. Especially in a more complicated social context as mentioned above, third-party decision makers, on the one hand, try to identify possible intentions underlying the negative acts committed by offenders. On the other hand, they also try to understand the affective state of victims harmed by the norm violation so that they exhibit empathic feelings for victims. A vast amount of fMRI and lesion studies revealed that the bilateral temporo-parietal junction (TPJ; more specifically, the ventral part^16^) plays a crucial role in people’s mentalizing ability^17^,^18^,^19^. Moreover, a recent pharmacological fMRI study further showed increased TPJ (esp. the left part) activity in third parties while seeing the victim being helped under intranasal oxytocin treatment^20^.

Besides the potential mentalizing process induced by other-regarding attention, cognitive control networks might be involved when making a specific choice running against the norm (i.e., fairness) concern addressed by a certain attention focus. For instance, third-party decision makers might need more cognitive resource while choosing to help the victim when focusing on the offender’s violation, as this requires overriding the more salient impulse to punish the offender which is more consistent with the retributive concern implied by focusing on offender’s violation. Previous neuroimaging studies showed stronger signals in regions including the inferior frontal gyrus extending to the anterior insula (IFG/AI; also labeled as ventral lateral prefrontal cortex) and dorsal anterior cingulate cortex (dACC) during control-relevant processes ranging from simple motor inhibition^21^ to complex contexts such as conflict monitoring^22^ and decision making^23^. Taken together, these findings indicate a modulatory effect of other-regarding attention on the TPJ as well as the control networks during third-party altruistic decision making.

To further elucidate the neural mechanisms underlying the modulatory effect of other-regarding attention on third-party altruistic decisions, we combined a modified third-party economic game paradigm with functional MRI (fMRI) in an incentivized context. As third-party decision maker, participants saw a series of (un-)fair monetary allocations between unknown proposers and recipients and were asked to determine whether to punish the offender (i.e., to decrease the payoff of the proposer) or help the victim (i.e., to increase the payoff of the recipient) using their own monetary endowment. Importantly, while making their decisions, participants were asked to consider the (un-)fairness of the offer made by the proposer (i.e., offender-focused block, OB), the feelings of the victim receiving the offer (i.e., victim-focused block, VB) or to make their choices naturally without a specific focus (i.e., baseline block, BB). Given the previous findings mentioned above, we expected help and punish proportions to be increased in VB and OB compared to BB. On the neural level, with specific focus on the bilateral TPJ, we hypothesized that higher activation in TPJ can be observed under other-regarding attention conditions (i.e., OB and VB) compared to BB, as more mentalizing processes (esp. intention inference) might be active while focusing on either the offender or the victim. Concerning the specific altruistic choice, we expected enhanced activation in TPJ induced by the other-regarding attention compared with BB. Finally, we hypothesized that regions associated with cognitive control or inhibition (i.e., IFG, dACC) are more active, especially when participants decide to help the victim in OB (vs. help in BB or VB).

## Results

### Behavioral Results

Participants in the MAIN sample reported much higher subjective feelings for unfairness during target offers with unequal monetary allocation between the offender and the victim than during offers with equal allocation (*t*(45) = 38.59, *p* < 0.001). This finding held true for the other subsamples (HELP subsample: *t*(41) = 36.00, *p* < 0.001; PUNISH subsample: *t*(21) = 24.52, *p* < 0.001; HELPUN subsample: *t*(19) = 23.22, *p* < 0.001; see Table S1 for details).

**Figure 1:**
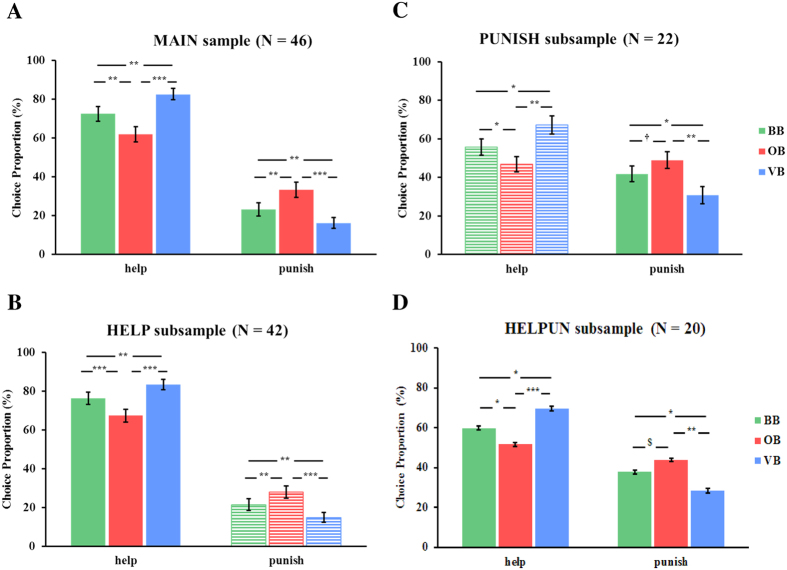
Proportion of altruistic choices in different other-regarding attention conditions. A pairwise comparison between the conditions was performed on help and punishment proportion for (**A**) the MAIN sample, (**B**) the HELP subsample, (**C**) the PUNISH subsample and (**D**) the HELPUN subsample. BB = baseline block, OB = offender-focused block, VB = victim-focused block; ^$^*p* < 0.1, ^†^*p* < 0.05; *LSD* correction; *p < 0.05, **p < 0.01, ***p < 0.001, *Bonferroni* correction. Shading patterns indicate the non-relevant decision type for the specific subsample. Error bars represent the SEM.

For choice proportion, the repeated measures ANOVA revealed a significant main effect of attention focus on help (*F*(2,90) = 21.10, *p* < 0.001, partial *η*^2^ = 0.32) and punishment choices (*F*(2,90) = 17.91, *p* < 0.001, partial *η*^2^ = 0.29) in the MAIN sample (see Fig. 1A). Concerning help choices, post-hoc pairwise comparison yielded a significant decrease of choice proportion in OB but an increase in VB, both compared to the BB (both *p* < 0.01, *Bonferroni* corrected). The effect was reversed for punishment choices: the choice proportion was higher in OB but lower in VB, both compared to the BB (both *p* < 0.01, *Bonferroni* corrected). The exhibited behavior was consistently seen in the HELP (help: *F*(2,82) = 26.06, p < 0.001, partial *η*^2^ = 0.39; punish: *F*(2,82) = 18.57, *p* < 0.001, partial *η*^2^ = 0.31; see Fig. 1B), the PUNISH subsample (help: *F*(2,42) = 12.96, *p* < 0.001, partial *η*^2^ = 0.38; punish: *F*(2,42) = 9.95, *p* = 0.001, partial *η*^2^ = 0.32; see Fig. 1C) as well as the HELPUN subsample (help: *F*(2,38) = 12.92, *p* < 0.001, partial *η*^2^ = 0.41; punish: *F*(2,38) = 9.30, *p* < 0.001, partial *η*^2^ = 0.33; see Fig. 1D and Table S2 for details).

For the mean decision time of help choices in the HELP subsample, the same analysis yielded a main effect of attention focus (*F*(2,82) = 17.23, *p* < 0.001, partial *η*^2^ = 0.30). Post-hoc pairwise comparison showed a longer decision time in the OB than that in the BB or VB (both *p* < 0.001, *Bonferroni* corrected). A marginal but non-significant main effect was found in the mean transfer amount of help choices (*F*(2,82) = 3.24, *p* = 0.065, partial *η*^2^ = 0.07). No significance was detected in neither the mean decision time nor the mean transfer amount of punishment choices in the PUNISH subsample (both *p* > 0.06). To be consistent with the GLM analysis (i.e., GLM1), we additionally ran the same analyses on mean decision time and mean transfer amount of all valid decisions regardless of specific choice type (i.e., help, punish and keep) in the MAIN sample. Similarly, the main effect of attention was detected in both analyses (mean decision time: *F*(2,90) = 25.78, *p* < 0.001, partial *η*^2^ = 0.36; mean transfer amount: *F*(2,90) = 4.03, *p* = 0.036, partial *η*^2^ = 0.08). Post-hoc pairwise comparison showed a longer decision time in the OB (vs. BB or VB; both *p* < 0.001, *Bonferroni* corrected) and a higher transfer amount in the VB (vs. BB or OB; both *p* < 0.05, *LSD* corrected). In the HELPUN subsample, a 3-by-2 repeated-measure ANOVA showed a main effect of attention (*F*(2, 38) = 3.75, *p* = 0.047, partial *η*^2^ = 0.17) and altruistic choice type (*F*(1, 19) = 5.84, *p* = 0.026, partial *η*^2^ = 0.23) and a trend-to-significant interaction effect (*F*(2, 38) = 2.94, *p* = 0.065, partial *η*^2^ = 0.13) on mean decision time. Post-hoc pairwise comparison showed a longer decision time in OB (vs. BB; *p* = 0.002, *Bonferroni* corrected) and for punishment choices (*p* = 0.026). However, neither the main effect nor the interaction on the mean transfer was significant (all *p* > 0.25; see Table 1 for details about mean decision time and mean transfer amount in all samples).

### Imaging Results

**Figure 2:**
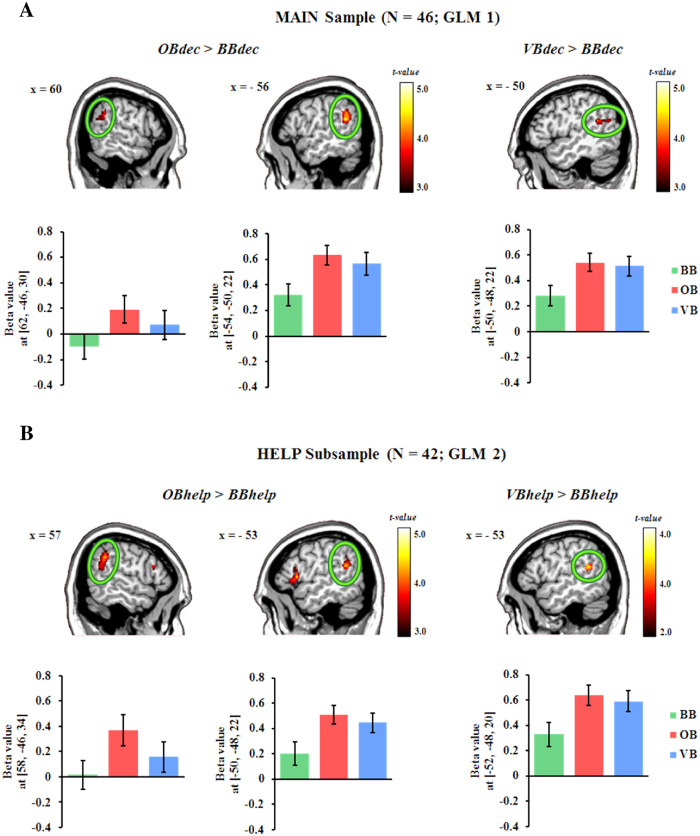
The effect of other-regarding attention on the bilateral TPJ. (**A**) *OBdec* > *BBdec* and *VBdec* > *BBdec* based on the MAIN sample (GLM1). (**B**) *OBhelp* > *BBhelp* and *VBhelp* > *BBhelp* based on the HELP subsample (GLM2). Display threshold for *VBhelp* > *BBhelp: p* < 0.005 (unc.), *k* = 50; display threshold for rest contrasts: *p* < 0.001 (unc.), *k* = 50. BB = baseline block, OB = offender-focused block, VB = victim-focused block; dec = decision; TPJ = temporo-parietal junction. The bar plots represent the beta values of the peak voxels (coordinates in parentheses). Error bars represent the SEM.

**Figure 3:**
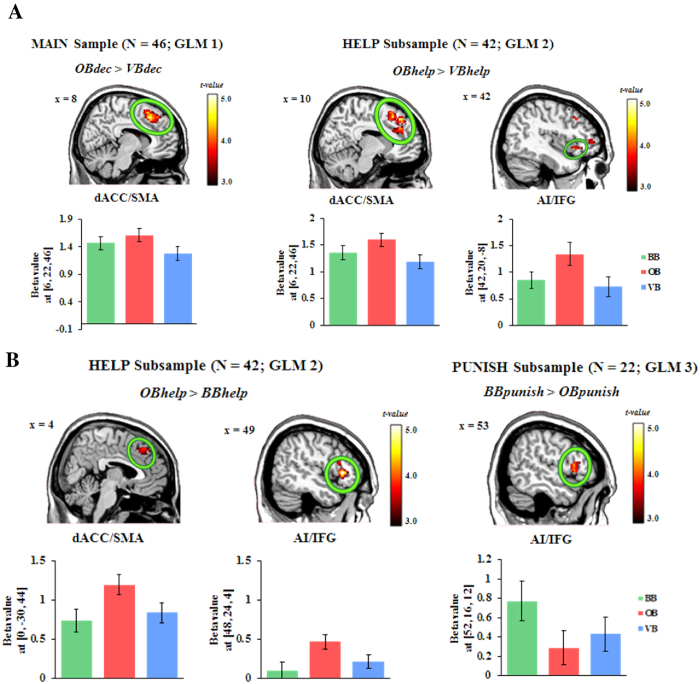
The effect of other-regarding attention on the control-relevant regions. **(A)** *OBdec* > *VBdec* based on the MAIN sample (GLM1) and *OBhelp* > *VBhelp* based on the HELP subsample (GLM2). (**B**) *OBhelp* > *BBhelp* based on the HELP subsample (GLM2) and *BBpunish* > *OBpunish* based on the PUNISH subsample (GLM3). Display threshold: *p* < 0.001 (unc.), *k* = 50. BB = baseline block, OB = offender-focused block, VB = victim-focused block; AI = anterior insula, dACC = dorsal anterior cingulate cortex, IFG = inferior frontal gyrus, SMA = supplementary motor area. The bar plots represent the contrast values of the peak voxels (MNI coordinates in parentheses). Error bars represent the SEM.

Based on the MAIN sample (GLM1), we observed an enhanced activation in the left and the right (peak MNI coordinates: 62/−46/30, t(90) = 3.93, p(SVC-FWE) = 0.046) TPJ of the contrast *OBdec* > *BBdec* (see Fig. 2A). The left TPJ (peak MNI coordinates: −50/−48/22, t(90) = 4.07, p(SVC-FWE) = 0.027) also showed a stronger response during decision making in VB (i.e., *VBdec* > *BBdec*; see Fig. 2A). Besides, the dACC extending to the supplementary motor area (SMA) was active during decision making in OB compared to VB (i.e., *OBdec* > *VBdec*; see Fig. 3A; see Table S3 for details of all activated regions).

In the HELP subsample (GLM2), the contrast *OBhelp* > *BBhelp* yielded a stronger activation in the bilateral TPJ (see Fig. 2B), the bilateral IFG extending to the AI together with the dACC/SMA (see Fig. 3B). We also observed a trend-to-significant increased activations in the left TPJ (peak MNI coordinates: −52/−48/20; *t*(82) = 3.67, *p*(SVC-FWE) = 0.089) in the contrast of *VBhelp* *>* *BBhelp* (see Fig. 2B). Besides, the dACC/SMA showed higher activation during help choices in OB compared to VB (i.e., *OBhelp* > *VBhelp*). We also observed a similar trend in the right IFG/AI (cluster-level *p*(WBC-FWE) = 0.058) in the same contrast (see Fig. 3A; see Table S4 for details of all activated regions).

Based on the PUNISH subsample (GLM3), we only found a trend-to-significant increased activation in the right IFG (cluster-level *p*(WBC-FWE) = 0.051) during punishment decisions in BB compared with OB (i.e., *BBpunish* > *OBpunish*, see Fig. 3B). No other regions were detected in other contrasts under the same threshold.

The analyses on the HELPUN subsample (GLM4) further revealed regions modulated by the interaction effect between attention focus and altruistic choice type. Particularly, the right IFG/AI showed higher activity during help (vs. punish) choices in OB (vs. BB). Moreover, the dACC/SMA extending to dorso-medial prefrontal cortex displayed stronger activity during help (vs. punish) choices in OB (vs. VB; see Figure S1; also see Table S5 for details of all activated regions).

## Discussion

The current fMRI study adopted a behavioral economic paradigm to investigate the modulatory effect of attention focus on third-party altruistic decision making in the context of social norm violations (i.e., unfairness). In general, our results confirmed the hypotheses that third-party participants’ altruistic decisions were biased by the imposed attention focus, which was mirrored by the involvement of neural circuitry that is closely associated with mentalizing (e.g., TPJ) and cognitive control processing (e.g., IFG/AI, dACC/SMA).

We first showed that participants as third-party decision makers were more prone to help when they were asked to focus on the feelings of the unfairly treated victims, whereas they did not only display less help choices but more punishment when asked to consider the (un-)fair action of the selfish offenders. Importantly, the similar attention-dependent choice pattern was also observed across different subsamples, which further indicates that this effect is robust to individual difference. In consistency with our findings, previous studies showed that attention played an active role in influencing complex decision making. For example, Hare *et al*. (2011) manipulated participants’ attention focus via exogenous cues towards either the healthiness or the taste of food items and found that non-dieting participants made healthier choices (i.e., participants were more likely to choose healthy but un-tasty foods) when asked to consider the healthiness of the food before making their choices^23^. In the social domain, two recent studies adopting a behavioral economic paradigm found similar results. By using a modified version of the dictator game, Hutcherson & Rangel (2014) revealed that focusing on either the ethics underlying a behavior (i.e., “consider the right thing to do”) or the empathy (i.e., “consider the partner’s feeling”) led the participants to exhibit a higher percentage of generous choices^24^. Makwana *et al*. (2014) employed a modified ultimatum game, in which participants, playing the role of recipients, rejected unfair offers provided by anonymous proposers more often if they were asked to consider its fairness before making a decision (i.e., fairness-focus condition). In contrast, participants were more likely to accept the offer, if their focus was directed to their own monetary payoff (i.e., money-focus condition)^25^. Moreover, Gromet & Darley (2009) have shown that manipulating the focus of a third-party observer towards different aspects within a hypothetical criminal context could influence the way to achieve justice^12^. Here, for the first time, we extend the modulatory effect of attention on decision making to third-party altruistic choices in an incentivized context.

We further investigated the neural mechanism underlying the modulatory effect of attention focus, with specific focus on the TPJ. In line with our hypotheses, the TPJ showed stronger activation during decision making in either OB or VB (vs. BB). A growing number of studies show that the TPJ (esp. the right side; but also see^19^) engages in tasks relevant to social cognition^26^,^27^, especially theory-of-mind (ToM)/mentalizing^17^,^18^ and empathic concern^28^,^29^.

Intriguingly, the TPJ (esp. the left TPJ) was also active when the third-party chose to help the victim in both OB and VB (vs. BB). Unlike altruistic punishment, choosing to help benefits others without causing any harm, rendering it as preferred choice of third-parties^8^,^20^ and being more rewarded as well as trusted by fourth-party observers^30^,^31^. Previous studies consistently showed the TPJ to be closely linked to moral decision making^32^,^33^ and altruistic behavior^34^, especially if these behaviors benefit others without causing harmful consequence. For instance, Morishima and colleagues detected a significant positive correlation between the grey matter volume of right TPJ and the degree of aversion to own more money than others^35^. In another study, the right TPJ not only showed higher activity when people made generous choices, but it also correlated with the difference of self-other monetary utility during generous decisions, which suggest its role in resisting the temptation to be selfish^36^. Taken together, our findings indicate that mentalizing and related processes are involved in other-regarding focus conditions (i.e., OB and VB), as reflected in the TPJ activation, which is more sensitive to compensatory behavior, a form of altruism only benefiting others.

However, the function of the TPJ is not limited to the social domain but also extends to more general cognitive processes such as attention reorientation^37^,^38^. To elucidate the functional heterogeneity within the (right) TPJ, Mars and colleagues (2012) further identified two sub-regions of the TPJ, namely the anterior and posterior part, by using diffusion-weighted tractography-based parcellation, and found different resting-state functional connectivity patterns of each sub-region^16^. Consistently, Bzdok and colleagues (2013) supported the functional difference between the anterior and posterior part of the right TPJ with multi-modal connectivity-based parcellation^39^. However, recent evidence by means of multivoxel pattern analysis suggested a functional convergence in the bilateral TPJ, as it was not only commonly activated by both attention and ToM-relevant tasks but also discriminated these tasks (esp. the right TPJ)^40^. Having been treated by continuous theta burst stimulation to inhibit the anterior part of the right TPJ, participants showed worse performance in both attention and ToM tasks, again indicating the functional convergence of TPJ^41^. Given the above evidence, the interpretation, which links TPJ activation to mentalizing processes, should be treated with cautions, as these results might also be interpreted as a reflection of domain-general attention level change induced by the manipulation.

Apart from the TPJ, regions relevant to cognitive control, including the IFG/AI and the dACC/SMA, also played an important role in the modulatory effect of attention focus on help and punishment choices. More importantly, our further investigation revealed the interaction effect between attention focus and altruistic choice type on both regions, indicating their crucial function in the potential mechanism underlying the attention-dependent choice-preference shift. As reported by numerous studies, the IFG/AI is one of the key components in the ventral attention network^42^,^43^ and further involved in cognitive control, such as response inhibition, task switching or memory retrieval inhibition^21^,^44^ (but also see^45^). Moreover, control-relevant activation of the IFG/AI can also be modulated by a different attention focus during decision making. For example, the IFG was found to exhibit higher activity when participants were asked to consider the healthiness of the food as mentioned above^23^, which indicates that attention cues increase cognitive control processes to reach the long-term goal (i.e., healthiness) over the short-term feature (i.e., taste). Likewise, our results showed that higher activities in the IFG/AI (esp. the right side) were observed during help choices in OB, accompanied by a lower help proportion compared to either BB or VB. Consistently, help choices were less frequently selected in OB. Similarly, a lower IFG/AI activation was found during punishment choices in OB (vs. BB), together with an increased likelihood to punish the offender. These two results also drove the interaction effect that higher IFG/AI activity was detected during help (vs. punishment) choice in OB, compared with the same contrast in BB. All above results implied that more (less) cognitive control is required for choosing to help (punish) in OB, as help (punishment) is incongruent (congruent) with the goal of OB.

Another critical region is the dACC/SMA, which exhibited stronger activity during help choice in OB compared with VB. Correspondingly, it also showed higher response during help (vs. punishment) choice in OB (vs. VB). A substantial number of literatures has shown that the dACC (or anterior middle part of the cingulate cortex) engages in functions related to cognitive control^46^, especially conflict monitoring and resolution^22^,^47^, which is associated with an increased demand on cognitive processing indexed by the reaction time. For example, in the Stroop task, a common paradigm to measure attention and conflict control, the dACC showed higher activity during incongruent trials (i.e., to name the color of the word “red” printed in green) associated to increased reaction time^48^,^49^. Intriguingly, the change in decision time due to attention focus appeared together with increased activations in the dACC/SMA in our study, especially when making help choices in OB, which is consistent with previous findings mentioned above.

Nevertheless, due to the potential problem of reverse inference^50^, we cannot completely rule out the alternative functions which might be exerted by AI/IFG as well as dACC/SMA during the decision process (same problem as for the interpretation of TPJ activation, see discussion above). For example, given the well-known role of AI and dACC in empathy for other’s pain or suffering^28^,^51^,^52^, the increased help-related activity of both regions in the OB (vs. BB) might also reflect stronger empathic concern for the victim while additionally considering the unfairness of the offender.

Several limitations to the current studies should be noted. First, the modulatory effect of attention focus in VB was not strong at the neural level. One possible explanation might be lower severity degree of norm violation of the present study. Previous studies of third-party punishment adopted severe criminal offenses (e.g., a person was robbed or even raped) as examples for violation^53^,^54^. In these cases, participants might recruit stronger mentalizing processes to understand the affective state of the victim, further enhanced by directing the attention to the feeling of the victim. However, victims in our case only received less money, which might not require additional cognitive resource to understanding their affective state even when people focused on the victim. As a consequence, it might blur the difference in TPJ activation between VB and BB. Second, altruistic choices of participants were unequally distributed across the three conditions. Specifically, participants in general preferred compensating behavior and rarely choose to punish, especially in VB. Consistent with our previous findings^8^,^20^, this result was also supported by a recent study showing that third-party decision makers, in a similar situation of fairness norm violation, favored less to punish the unfair offender if they could also have the chance to help the victim at the same time^31^. As a consequence, low number of trials is insufficient to warrant a stable estimation of the fMRI-BOLD signal for the respective conditions. Thus we had to divide our sample into three sub-samples so that we could ensure a sufficient amount of help choices, punishment choices or both choices in each attention condition while keeping a fairly large sample size. Since our choice-specific analyses were performed on different and reduced samples, it limits the generalization of our findings.

To conclude, our fMRI study revealed that third-party altruistic choices can be modulated by directing their attention focus to other-regarding aspects. Moreover, we further characterized the neural basis potentially underlying this effect, in particular the active involvement of the TPJ as well as the regions relevant to cognitive control (esp. AI/IFG, dACC/SMA). Our findings have useful implications in understanding the cognitive and neural bases underlying complex social decision making such as judicial judgment, where judgements may be altered by the focus laid on either the victim or the offender.

## Methods

### Participants

Fifty participants attended our fMRI experiment (23 male; mean age = 24.6, SD = 3.5; 4 left handedness) and were recruited via online flyers at the University of Bonn and from the local community. All participants were free of medication, reported no history of neurological or psychiatric disorders, and had normal (or corrected-to-normal) vision as well as color perception. The study was approved by the ethics committee of the University of Bonn and written informed consent was received from all participants according to the Declaration of Helsinki (BMJ 1991; 302: 1194). All experimental protocols and procedures were conducted in accordance with the IRB guidelines for experimental testing and were in compliance with the latest revision of the Declaration of Helsinki.

### Stimuli and Design

Stimuli of the present fMRI task included 126 pairs of unfair monetary allocations with different payoff combinations, similar to those used in previous studies (for similar procedure, see Leliveld *et al*., 2012, and Hu *et al*., 2015) but with the following modifications. First, we only selected offers in which the offender’s payoff was more than twice the victim’s payoff, aiming to increase the motivation for altruistic decisions as shown in previous literature^2^. Second, we added a randomized fluctuation to the integer of the payoff to further increase the variation of the stimuli to maintain participants’ attention during the experiment. In detail, seven different combinations of monetary allocations (meeting the first requirement) were selected as template offers (i.e., total payoff 9 €: 7/2, 8/1; total payoff 10 €: 7/3, 8/2, 9/1; total payoff 11 €: 8/3, 9/2; the first number refers to the offender’s payoff and the second to the victim’s payoff). Here, a random value ranging from 0 to 0.2 was added to or subtracted from the offender’s payoff for each template. The victim’s payoff was then determined by subtracting the offender’s payoff from the total sum of that template (e.g., if the template allocation was 7/2, the displayed offender’s payoff could finally become any value between 6.80 € and 7.20 €, such as 7.01 €; thus the victim’s payoff was 1.99 €, namely 9 € minus 7.01 €). Finally, the payoff of both parties was always below 10 €, to avoid the confounding effect of attention shift driven by an unequal amount of digits.

To increase the credibility of the experimental context, we also added 18 pairs of fair monetary allocation with different payoff combinations. Similar to unfair offers, the final payoff for fair offers was based on three templates (i.e., 4.5/4.5, 5/5, 5.5/5.5) and finally determined by modifying the integer with a random value ranging from 0 to 0.05 (e.g., if the template allocation was 4.5/4.5, the displayed offender’s payoff could finally become any value between 4.50 € and 4.55 €, such as 4.52 €; thus the victim’s payoff was 4.48 €, namely 9 € minus 4.52 €). Taken together, each of the 144 pairs of monetary allocation was presented once during the whole experiment (see Table S6 for details).

A mixed fMRI design was adopted for the present study with one factor (i.e., other-regarding attention; three levels: BB, OB, and VB). The fMRI session consisted one run, which included 18 blocks equally assigned to three conditions (6 blocks per condition): BB, OB, and VB. The blocks were fully randomized for each subject with the constraint of not more than three consecutive blocks belonging to the same condition. Each block included eight trials consisting of seven trials presenting unfair offers and one trial presenting a fair offer. Importantly, we designed the payoff structure in such a way that the average total payoff for all unfair offers within each block was the same (i.e., 10 €), to further control for the potential confounding effect due to the unequal payoff sums. The order of trials within each block was also fully randomized.

### Procedure

Before the day of scanning, participants completed online questionnaires assessing their demographics and personality characteristics. On the day of scanning, participants were first given the instructions about the experimental task. Participants were informed about another fictitious behavioral experiment, in which a separate group of participants were randomly assigned to the role of the offender (i.e., first-party; labeled as “Player A”) and the victim (i.e., second-party; labeled as “Player B”) in a dictator game. Each offender in that game received a specific amount of endowment and could freely distribute the money between him-/herself and the victim. Importantly, participants were told that decisions of the offenders would be used as stimuli in the current fMRI study. Participants as third-parties would receive 10 € as endowment in every round and could influence the final payoff of either the offender or the victim investing their own money with a cost ratio of 1:3^2^,^7^,^8^, meaning that by sacrificing 1 € of their own endowment they could either decrease the offender’s payoff by 3 € (i.e., punish) or increase the victim’s payoff by 3 € (i.e., help). To make sure decisions were made voluntarily, participants were also told that it was possible to keep their full endowment in the task. Also, they were told that one trial would be randomly selected and the payoff in that trial would be used as their final payment (i.e., 10 € minus the amount the participant transferred). They were told that their decisions might also be pay-off relevant for the corresponding offender (i.e., his/her original payoff minus triple the amount the participant transferred after choosing to punish) and the corresponding victim (i.e., his/her original payoff plus triple the amount the participant transferred after choosing to help), respectively. To enhance involvement in the task, participants were further instructed that the endowment per trial (i.e., 10 €) would be lost if they failed to show any response in that trial.

After participants passed a quiz to confirm their understanding of the task and finished practice rounds to familiarize with the paradigm, they were provided with the second part of the instruction elaborating the manipulation of attention focus. Here, participants were instructed to make decisions under three conditions in different blocks. In the OB, they were asked to consider the (un-)fairness of the offender’s proposal before making a decision, whereas in the VB, they were asked to consider the victim’s feeling receiving the unfair offer before making a decision. In the BB, participants were asked to make their decisions naturally without considering any specifically defined aspect. Importantly, to rule out possible confounding demand characteristics, participants were explicitly told that they should make the decisions they would prefer, independent of the attention condition^23^. Afterwards, participants completed another practice round inside the scanner, identical to the final experiment procedure.

**Figure 4:**
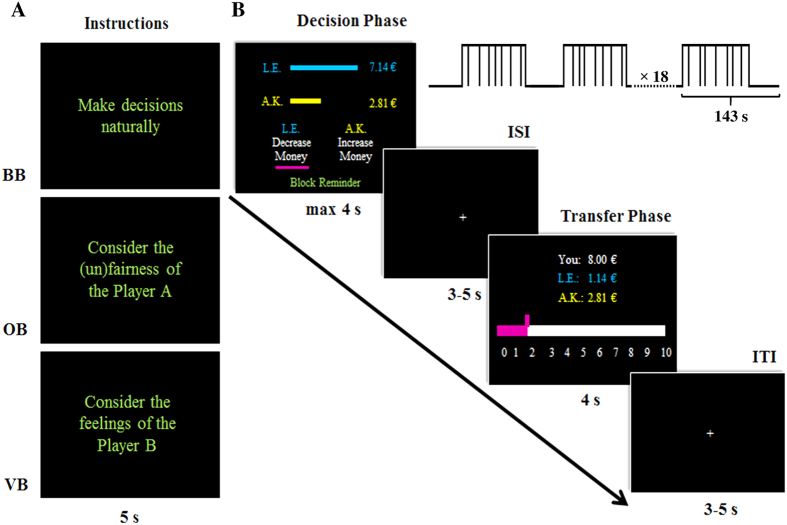
fMRI paradigm. (**A**) Before each block, an instruction slide to manipulate subjects’ attention focus was presented (from top to bottom: condition of BB, OB, VB; the offender was labeled as Player A, the victim was labeled as Player B). (**B**) Each trial starts with the decision phase in which subjects could choose to either costly decrease the offender’s (as shown in this case) or increase the victim’s monetary payoff. After a jittered ISI, subjects were asked to indicate how much of their own endowment they would like to sacrifice in order to change the respective money allocation (transfer phase). A jittered ITI completed the trial. BB = baseline block, OB = offender-focused block, VB = victim-focused block, ISI = inter-stimulus interval, ITI = inter-trial interval.

The MRI session comprised of a functional scan, followed by a structural scan. During the fMRI scanning, a 5 s instruction initializing one of the three attention-conditions (i.e., BB, OB or VB) was presented. This was followed by a block including 8 trials and a 15 s inter-block interval of fixation cross, resulting in a fixed block-duration of 143 s. Specifically, each trial began with the decision phase, which displayed one money allocation. Here, the information of the offender (i.e., the initials as well as the payoff) was always located in the upper position in blue, whereas the corresponding information of the victim was located in the lower position in yellow. The two options (i.e., decrease the payoff of the offender or increase the payoff of the victim) were displayed underneath the allocation. Importantly, their positions were counterbalanced across trials. Participants had maximally 4 s to respond by pressing a button with their left or right index fingers. Their choice was indicated by a purple line underneath the relevant option once the button was pressed. The decision phase was subsequently followed by an inter-stimulus interval (ISI) showing a fixation cross with a jittered duration of 3–5 s. To control for the trial duration, the remaining time of the decision phase (i.e., 4 s minus the decision time) was added to the ISI. This fixation was followed by a 4 s transfer phase. Here, participants could indicate how much of their own endowment they want to sacrifice according to their preceding decision. This choice was made by moving the cursor in steps of 0.5 €, again via pressing the button with their left or right index finger. The payoffs of all three parties were displayed and updated with the movement of the cursor. Moreover, the starting position of the cursor was randomized across trials. The transfer phase was followed by an inter-trial interval (ITI) showing another fixation cross with a jittered duration of 3–5 s (for trial procedure, see Fig. 4). If participants failed to respond within 4 s or made an unrealistically fast decision (i.e., decision time <200 ms), a 4s-screen, noting those behaviors, was presented instead of the transfer phase. All stimuli were presented using Presentation v14 (Neurobehavioral Systems Inc., *Albany, CA, USA*) on a 32″ liquid crystal display (NordicNeuroLab, *Bergen, Norway*) outside the scanner with a resolution of 800 × 600 pixels, using a mirror system attached to the head coil. Participants’ responses were collected via an MRI-compatible response device (NordicNeuroLab, *Bergen, Norway*).

Apart from the above described specifications, it is necessary to address further methodological details important to our paradigm. First, the words “help”, “punish”, “offender”, “victim” and “dictator game” were not used in the instructions (i.e., “increase”, “subtract” “player A”, “player B” and “money allocation game” were adopted instead) to avoid demand characteristics. Second, the offender could never lose money due to the punishment by the participant; that is to say, the minimum payoff for the offender was 0 €.

After scanning, participants completed a self-paced computer-aided rating task, in which they were asked to evaluate the same money allocations they already saw in the scanner on a 9-point Likert scale according to their subjective feeling of unfairness (0 = not at all, 8 = very much). In the end, participants received, via bank transfer, a 10 € show-up fee, a 5 € bonus for limiting their head motion during fMRI scanning (which, if exceeding 3 mm, would not be paid), and an extra payoff depending on their decision during the chosen trial (maximally 10 €).

### Data Acquisition

The imaging data was collected via a 3-Tesla Siemens Trio MRI system (Siemens, *Erlangen, Germany*), equipped with a 32-channel head coil at the Department of Epileptology, University Hospital Bonn. The functional imaging data was acquired using a T2*-weighted echo planar imaging (EPI) pulse sequence employing a BOLD contrast (TR = 2500 ms, TE = 30 ms, flip angle = 90°) in 37 axial slices (FOV = 192 × 192 mm^2^, matrix = 96 × 96, thickness = 3 mm, in-plane resolution = 2 × 2 mm^2^) covering the whole brain volume. Slices were axially oriented along the AC-PC plane and acquired in an ascending order. For later realignment and normalization, a high-resolution structural T_1_-weighted image was recorded for every subject using a 3D MRI sequence (TR = 1660 ms, TE = 2.75 ms, flip angle = 9°, matrix = 320 × 320, slice thickness = 0.8 mm, FOV = 256 × 256 mm^2^).

### Data Analysis

Four out of scanned 50 participants were excluded from the analyses due to either quitting the experiment (N = 1) or excessive head motion (i.e., >3 mm; N = 3). The data of 46 participants was finally adopted for further analyses (i.e., the MAIN sample; 21 males). To further investigate the effect of attention focus on help or punishment choice respectively or its interaction with the altruistic choice type (i.e., help or punish), we divided the MAIN sample into three sub-samples based on their behavior: 1) the HELP subsample (N = 42; 21 males) consisted of participants that exhibited at least 5 help choices (transfer amount > 0) in each of the three conditions (i.e., BB, OB and VB); 2) the PUNISH subsample (N = 22; 11 males) consisted of participants that showed at least five punishment choices (transfer amount > 0) in each of the three conditions; 3) the HELPUN subsample (N = 20; 10 males) consisted of participants that showed at least five help and punishment choices in each of the three conditions. The criterion of five trials was set given the stable parameter estimates of the BOLD signal while keeping a reasonable sample size to obtain sufficient statistical power^20^.

Behavioral analyses were conducted using SPSS 22 (IBM Corporation, *Armonk, NY, USA*). All reported p-values were two-tailed and *p* < 0.05 was considered statistically significant. The proportion of help and punishment choices of each condition was analyzed separately for all three subsamples. Mean decision time and mean transfer amount of money in each condition of help choices were only analyzed in the HELP subsample, whereas those of punishment choices were only analyzed in the PUNISH subsample, as some participants of the HELP subsample showed no punishment choices at all (i.e., decision time and transfer amount were not available in these cases) and vice versa. To examine the main effect of other-regarding attention on these dependent variables, a repeated measure one-way ANOVA was applied. To further test the interaction effect between attention focus and altruistic choice type on mean decision time as well as mean transfer amount in the HELPUN subsample, a 3-by-2 repeated measure ANOVA (i.e., factor 1: attention focus, BB/OB/VB; factor 2: altruistic choice: help/punishment) was applied. The assumption of sphericity was tested by Mauchly’s sphericity test and, if violated, a Greenhouse-Geisser correction was applied. To further disentangle the main and interaction effect, a post-hoc t-test was employed using a Bonferroni correction to control for multiple comparisons. In addition, a pairwise t-test was adopted on the post-scanning rating task to check whether the target offers with unequal monetary allocation can elicit stronger unfairness feeling compared to filter offers with equal allocation.

Functional imaging data was analyzed using SPM 8 (Wellcome Trust Centre for Neuroimaging, University College London, *London, UK*). The preprocessing of the functional data followed the common pipeline: 1) for each participant, the first 3 volumes were discarded to allow for the stabilization of the BOLD signal; 2) EPI images were realigned to the first volume to correct motion artifacts and then corrected for slice timing; 3) the structural T_1_ image was co-registered to the mean EPI images and then segmented into white-matter, grey-matter and cerebrospinal fluid to generate normalization parameters to MNI space; 4) all EPI images were normalized to the MNI space, resampled with a 2 × 2 × 2 mm^3^ resolution, based on parameters generated in the previous step, and then smoothed using an 8-mm isotropic full width half maximum (FWHM) Gaussian kernel; 5) high-pass temporal filtering was performed with a cut-off value of 286 s to model the block effect (i.e., twice the block duration).

### General Linear Model (GLM) analyses

On the single-subject level, four different GLMs convolved with the canonical HRF were applied to each sample. GLM1, aimed to test the main effect of other-regarding attention on general decision processing regardless of the specific choice type, was applied to the MAIN sample. In particular, GLM1 included three regressors of interest, namely onsets of stimuli presentation during valid decision (regardless of specific choice, i.e., help, punish and keep) in BB, OB and VB (i.e., *BBdec, OBdec, VBdec*; duration equals the decision time). Besides, GLM1 included 6 regressors modeling events of no interests, namely 1–3) onsets of BB, OB, and VB blocks (duration equals 143 s; the period from the offset of the BB instruction to the onset of the instruction of the next block), 4) onsets of all transfer phases (duration equals 4 s), 5) onsets of all instructions (duration equals 5 s) and 6) onsets of stimuli presentation during invalid decision phases (i.e., no response trials, duration equals the 4 s; trials with a decision time less than 200 ms or fair offers, duration equals the decision time).

GLM2 aimed to detect the other-regarding attention effect on neural correlates for help choices, which was applied on the HELP subsample. GLM2 consisted of three regressors of interest, namely onsets of stimuli presentation during help choices in BB, OB and VB (i.e., *BBhelp OBhelp VBhelp*; duration equals the decision time). The rest of the regressors were equivalent to those in GLM1, except that onsets of stimuli presentation during keep and punishment choices (duration equals the decision time) were considered as onsets of invalid decisions.

GLM3 aimed to detect the other-regarding attention effect on neural correlates of punishment choices, which was applied to the PUNISH subsample. GLM 3 consisted of three regressors of interest, namely onsets of punishment choices in BB, OB and VB (i.e., *BBpunish, OBpunish, VBpunish*; duration equals the decision time). The rest of the regressors were equivalent to those in GLM1, except that onsets of stimuli presentation during keep as well as help choices (duration equals the decision time) were considered as onsets of invalid decisions.

GLM4 aimed to detect the interaction between the other-regarding attention effect and altruistic choice type, which could further indicate the potential neural mechanism underlying the choice-preference shift with different attention foci. GLM4 was applied to the HELPUN subsample and consisted of six main regressors of interest, namely onsets of both help and punishment choices in BB, OB and VB (i.e., *BBhelp, OBhelp, VBhelp, BBpunish, OBpunish, VBpunish*; duration equals the decision time). The rest of the regressors were equivalent to those in GLM1, except that onsets of stimuli presentation during keep choices (duration equals the decision time) were considered as onsets of invalid decisions.

For all GLMs, the six movement parameters were added to the model to account for head motions. Individual contrasts of these regressors of interest were built in each GLM (i.e., GLM1: *BBdec, OBdec, VBdec*, vs. implicit baseline respectively; GLM2: *BBhelp, OBhelp, VBhelp* vs. implicit baseline respectively; GLM3: *BBpunish, OBpunish, VBpunish* vs. implicit baseline respectively; GLM4: *BBhelp* vs. *BBpunish, OBhelp* vs. *OBpunish, VBhelp* vs. *VBpunish* respectively).

On the group level, individual contrasts were forwarded to a one-way flexible factorial ANOVA model, in which we performed comparisons by pair-wise t-tests between different conditions based on the corresponding samples. We adopted a whole-brain corrected (WBC) threshold of *p* < 0.05 at the cluster-level controlling for family-wise error (FWE) rate with an uncorrected voxel-level threshold of *p* < 0.001 as the cluster-defining threshold^55^ for results in GLM1–3. Given the smaller sample size (N = 20) of GLM4, a more lenient uncorrected voxel-level threshold of *p* < 0.001 was adopted. Additionally, a small volume correction (SVC) was conducted within pre-defined structural-based mask of the bilateral TPJ^34^, only when the results in TPJ did not survive the whole-brain correction.

For all analyses, regions were labelled according to the automated anatomical labelling (AAL) template via the xjView toolbox (http://www.alivelearn.net/xjview8/). Furthermore, parameter estimates of the peak voxels (i.e., beta values) were extracted using the MarsBaR toolbox (http://marsbar.sourceforge.net/) and visualizations of fMRI results were realized via MRIcron (http://www.mccauslandcenter.sc.edu/mricro/mricron/).

## Additional Information

**How to cite this article:** David, B. *et al*. Other-regarding attention focus modulates third-party altruistic choice: An fMRI study. *Sci. Rep.* **7**, 43024; doi: 10.1038/srep43024 (2017).

**Publisher's note:** Springer Nature remains neutral with regard to jurisdictional claims in published maps and institutional affiliations.

## Supplementary Material

Supporting Information

## Figures and Tables

**Table 1:** Mean (±SD) of decision time and transfer amount in corresponding subsamples respectively during scanning.

		BB	OB	VB
***All valid choices of MAIN sample (N = 46; GLM1)***				
Decision Time (ms)		1562.12 (386.98)	1736.47 (398.72)	1563.49 (402.20)
Transfer Amount (€)		2.28 (1.28)	2.30 (1.27)	2.50 (1.35)
***Help choices of HELP subsample (N = 42; GLM2)***				
Decision Time (ms)		1571.22 (399.38)	1731.18 (438.56)	1569.66 (416.95)
Transfer Amount (€)		2.28 (1.28)	2.30 (1.27)	2.50 (1.35)
***Punishment choices of PUNISH subsample (N = 22; GLM3)***				
Decision Time (ms)		1814.82 (364.88)	1901.08 (368.29)	1945.22 (363.91)
Transfer Amount (€)		2.09 (0.89)	2.12 (0.62)	2.26 (1.05)
***Help and punishment choices of HELPUN subsample (N = 20; GLM4)***				
Decision Time (ms)	*Help*	1800.85 (375.72)	1913.37 (418.93)	1778.45 (420.36)
	*Punishment*	1844.99 (366.26)	1934.31 (360.70)	1958.63 (379.40)
Transfer Amount (€)				
	*Help*	2.13 (1.00)	2.18 (1.22)	2.44 (1.39)
	*Punishment*	2.16 (0.91)	2.15 (0.63)	2.30 (1.07)

Note: BB = baseline block, OB = offender-focused block, VB = victim-focused block.
