# Supplementary material for: Other-regarding attention focus modulates third-party altruistic choice: An fMRI study
Source: Sci Rep. 2017 Feb 21;7:43024. doi: 10.1038/srep43024 (PMC5318960; doi:10.1038/srep43024)
Supplement: Supporting Information [file srep43024-s1.pdf]

Other-regarding attention focus modulates third-party altruistic choice: An fMRI study

Bastian David[1,2,†], Yang Hu[1,†,*], Frank Krüger[3], Bernd Weber[1,2]

[1]Center for Economics and Neuroscience, University of Bonn, 53127, Germany

[2]Department of Epileptology, University Hospital Bonn, 53127, Germany

[3]Department of Psychology, George Mason University, VA 22030, USA

[†]These authors contributed equally to this work.

[*]Corresponding: Yang Hu, Center for Economics and Neuroscience, University of Bonn, Nachtigallenweg 86, Bonn, 53127, Germany. Email: huyang@uni-bonn.de

Supplementary Information

Supplementary Table S1 Mean (± SD) of unfairness rating after scanning

	Target offer with unequal monetary allocation	Filter offer with equal monetary allocation
MAIN sample (N = 46; GLM1)	5.95 (0.60)	0.54 (0.80)
HELP subsample (N = 42; GLM2)	5.97 (0.62)	0.57 (0.82)
PUNISH subsample (N = 22; GLM3)	5.92 (0.58)	0.54 (0.62)
HELPUN subsample (N = 20; GLM4)	5.91 (0.59)	0.54 (0.63)

Note: Rating scores were collected on a 9-point Likert scale in terms of participants' subjective unfairness feeling (0 = not at all, 8 = very much); BB = baseline block, OB = offender-focused block, VB = victim-focused block.

Supplementary Table S2 Mean (± SD) of altruistic choice proportion (%) during scanning

	help			punish		
	**BB**	**OB**	**VB**	**BB**	**OB**	**VB**
MAIN sample	72.57	61.69	82.45	23.19	33.44	15.99
(N = 46; GLM1)	(25.99)	(26.86)	(21.22)	(23.33)	(26.73)	(20.44)
HELP subsample	76.36	67.35	83.44	21.43	28.00	14.85
(N = 42; GLM2)	(20.23)	(20.99)	(17.65)	(19.45)	(20.25)	(16.49)
PUNISH subsample	55.75	46.86	67.21	41.88	48.91	30.85
(N = 22; GLM3)	(19.47)	(18.84)	(21.88)	(19.11)	(20.27)	(20.96)
HELPUN subsample	59.77	51.55	69.53	37.74	43.81	28.34
(N = 20; GLM4)	(14.36)	(11.74)	(16.21)	(13.24)	(12.32)	(14.43)

Note: BB = baseline block, OB = offender-focused block, VB = victim-focused block.

Supplementary Table S3 Pairwise contrast of neural correlates during decision making regardless of choice type between different attention conditions in MAIN sample (N = 46; GLM1)

Brain Region	Hemisphere	Cluster Size	MNI Coordinates x	y	z	BA	T-value
OBdec vs. BBdec							
Temporo-Parietal Junction	L	508	-54	-50	22	40	4.71*
Temporo-Parietal Junction	R	126	62	-46	30	40	3.93†
Inferior Frontal Gyrus/ Anterior Insula	L	114	-46	30	-6	47	4.14
Precentral Gyrus	L	274	-42	2	58	6/8	4.52*
BBdec vs. OBdec							
No cluster							
VBdec vs. BBdec							
Temporo-Parietal Junction	L	165	-50	-48	22	40	4.07†
BBdec vs. VBdec							
No cluster							
OBdec vs. VBdec							
Dorsal Anterior and Mid-Cingulate Cortex/ Supplementary Motor Area	B	626	6	22	46	6/8/32	5.00*
Thalamus/Caudate/Lateral Ventricle	B	194	-2	-2	16		4.95*
VBdec vs. OBdec							
No clusters							

Note: Regions shown here met the uncorrected voxel-level threshold of $p < 0.001$ with $k = 100$; dec = decision, BB = baseline block, OB = offender-focused block, VB = victim-focused block, L = left, R = right, B = bilateral, BA = Brodmann Area; brain regions are labeled according to the automated anatomic labeling template.

*Significant at $p < 0.05$ family wise error (FWE) rate corrected at the cluster level

†Significant at $p < 0.1$ family wise error (FWE) rate corrected at the cluster level

Supplementary Table S4 Pairwise contrast of neural correlates during help choice between different attention conditions in HELP subsample (N = 42; GLM2)

Brain Region	Hemisphere	Cluster Size	MNI Coordinates			BA	T-value
			x	y	z		
OBhelp vs. BBhelp							
Inferior Frontal Gyrus	L	217	-54	16	6	45/47	4.46*
Anterior Insula	L	141	-28	18	-6	13	4.54†
Inferior Frontal Gyrus/ Anterior Insula	R	420	48	24	4	13/45/47	5.26*
Precentral Gyrus/ Middle Frontal Gyrus	L	291	-44	12	46	6/8	4.40*
Middle Frontal Gyrus	R	128	38	26	38	9	4.26
Dorsal Anterior and Mid-Cingulate Cortex/ Supplementary Motor Area	B	173	0	30	44	6/8/9	4.11*
Medial/Superior Frontal Gyrus	R	115	12	6	64	6/8/9	4.04
Temporo-Parietal Junction	L	191	-50	-48	22	40	4.58*
Temporo-Parietal Junction/ Inferior Parietal Lobule	R	323	58	-46	34	40	4.24*
BBhelp vs. OBhelp							
No cluster							
VBhelp vs. BBhelp							
No cluster							
BBhelp vs. VBhelp							
No cluster							
OBhelp vs. VBhelp							
Inferior Frontal Gyrus/ Anterior Insula	R	161	42	20	-8	13/45/47	4.28†
Inferior/Middle Frontal Gyrus	R	118	38	46	6	10	4.50
Dorsal Anterior and Mid-Cingulate Cortex/ /Supplementary Motor Area	B	1104	6	22	46	6/8/9/32	5.13*
Inferior Parietal Lobule	R	214	54	-50	42	40	4.42*
Caudate/Lateral Ventricle	B	191	-4	-2	16		4.38*
VBhelp vs. OBhelp							
No clusters							

Note: Regions shown here met the uncorrected voxel-level threshold of p < 0.001 with k = 100; BB = baseline block, OB = offender-focused block, VB = victim-focused block, L = left, R = right, B = bilateral, BA = Brodmann Area; brain regions are labeled according to the automated anatomic labeling template.

*Significant at $p < 0.05$ family wise error (FWE) rate corrected at the cluster level

†Significant at $p < 0.1$ family wise error (FWE) rate corrected at the cluster level

Supplementary Table S5 Pairwise contrast of neural correlates of help vs. punishment choice between different attention conditions in HELPUN subsample (N = 20; GLM4)

Brain Region	Hemisphere	Cluster Size	MNI Coordinates			BA	T-value
			x	y	z		
OB(help-punish)* vs. *BB(help-punish)							
Inferior Frontal Gyrus/ Anterior Insula	R	130	48	20	14	13/44/45/47	4.42
BB(help-punish)* vs. *OB(help-punish)							
No cluster							
VB(help-punish)* vs. *BB(help-punish)							
No cluster							
BB(help-punish)* vs. *VB(help-punish)							
No cluster							
OB(help-punish)* vs. *VB(help-punish)							
Superior Frontal Gyrus/ Supplementary Motor Area/Mid-Cingulate Cortex/	B	260	18	14	48	6/8	4.83*
VB(help-punish)* vs. *OB(help-punish)							
No cluster							

Note: Regions shown here met the uncorrected voxel-level threshold of $p < 0.001$ with k = 100; BB = baseline block, OB = offender-focused block, VB = victim-focused block, R = right, B = bilateral, BA = Brodmann Area; brain regions are labeled according to the automated anatomic labeling template.

*Significant at $p < 0.05$ family wise error (FWE) rate corrected at the cluster level

Supplementary Table S6 Stimuli used in the fMRI task

Block No.	Offer Type	Payoff	
		Offender	*Victim*
1	fair	4.53	4.47
1	unfair	6.83	3.17
1	unfair	6.98	2.02
1	unfair	7.94	1.06
1	unfair	8.00	2.00
1	unfair	8.04	2.96
1	unfair	8.92	2.08
1	unfair	9.19	0.81
2	fair	5.00	5.00
2	unfair	6.98	3.02
2	unfair	7.17	1.83
2	unfair	7.98	1.02
2	unfair	8.02	1.98
2	unfair	8.11	2.89
2	unfair	8.97	2.03
2	unfair	9.02	0.98
3	fair	5.55	5.45
3	unfair	6.81	2.19
3	unfair	6.84	3.16
3	unfair	7.96	3.04
3	unfair	8.07	0.93
3	unfair	8.16	1.84
3	unfair	9.01	1.99
3	unfair	9.03	0.97
4	fair	5.53	5.47
4	unfair	6.89	3.11
4	unfair	7.04	1.96
4	unfair	7.86	2.14
4	unfair	8.03	0.97
4	unfair	8.09	2.91
4	unfair	8.83	2.17
4	unfair	8.89	1.11
5	fair	4.54	4.46
5	unfair	6.97	3.04
5	unfair	7.10	1.90
5	unfair	7.91	2.09
5	unfair	7.92	1.08
5	unfair	8.20	2.80
5	unfair	8.99	1.01
5	unfair	9.13	1.87
6	fair	5.01	4.99

6	unfair	7.01	2.99
6	unfair	7.14	1.86
6	unfair	7.82	1.18
6	unfair	8.13	1.87
6	unfair	8.18	2.82
6	unfair	8.82	1.18
6	unfair	9.19	1.81
7	fair	5.50	5.50
7	unfair	6.85	3.15
7	unfair	6.97	2.03
7	unfair	7.88	3.12
7	unfair	7.94	2.06
7	unfair	8.17	0.83
7	unfair	8.99	2.01
7	unfair	9.13	0.87
8	fair	5.03	4.97
8	unfair	7.11	2.89
8	unfair	7.11	1.89
8	unfair	7.89	2.11
8	unfair	8.06	0.94
8	unfair	8.17	2.83
8	unfair	8.87	2.13
8	unfair	8.97	1.03
9	fair	5.04	4.96
9	unfair	6.89	2.11
9	unfair	7.16	2.84
9	unfair	7.90	3.10
9	unfair	7.98	2.02
9	unfair	8.14	0.86
9	unfair	8.82	2.18
9	unfair	9.01	0.99
10	fair	5.51	5.49
10	unfair	6.90	2.10
10	unfair	7.09	2.91
10	unfair	7.83	1.17
10	unfair	7.93	2.07
10	unfair	8.16	2.84
10	unfair	8.84	2.16
10	unfair	9.14	0.86
11	fair	4.55	4.45
11	unfair	7.05	1.95
11	unfair	7.05	2.95
11	unfair	7.95	2.05
11	unfair	8.10	2.90
11	unfair	8.12	0.88
11	unfair	8.84	1.16

11	unfair	9.04	1.96
12	fair	5.05	4.95
12	unfair	6.93	2.07
12	unfair	7.12	2.88
12	unfair	7.90	1.10
12	unfair	8.03	2.97
12	unfair	8.12	1.88
12	unfair	9.10	1.90
12	unfair	9.17	0.83
13	fair	4.50	4.50
13	unfair	6.86	2.14
13	unfair	6.94	3.07
13	unfair	7.81	1.19
13	unfair	7.89	3.11
13	unfair	8.05	1.95
13	unfair	9.09	0.91
13	unfair	9.09	1.91
14	fair	5.02	4.98
14	unfair	7.00	2.00
14	unfair	7.13	2.87
14	unfair	7.96	1.04
14	unfair	8.05	2.95
14	unfair	8.17	1.83
14	unfair	8.90	1.10
14	unfair	9.12	1.88
15	fair	4.52	4.48
15	unfair	7.13	1.87
15	unfair	7.18	2.82
15	unfair	7.86	1.14
15	unfair	7.99	3.01
15	unfair	8.18	1.82
15	unfair	8.87	1.13
15	unfair	8.88	2.12
16	fair	4.51	4.49
16	unfair	6.96	2.04
16	unfair	7.19	2.81
16	unfair	7.82	2.18
16	unfair	8.00	3.00
16	unfair	8.02	0.98
16	unfair	9.16	0.84
16	unfair	9.20	1.80
17	fair	5.54	5.46
17	unfair	7.15	2.85
17	unfair	7.19	1.81
17	unfair	7.84	3.16
17	unfair	7.97	1.03

17	unfair	8.20	1.80
17	unfair	9.05	1.95
17	unfair	9.08	0.92
18	fair	5.52	5.48
18	unfair	6.96	3.04
18	unfair	7.09	1.91
18	unfair	7.82	3.18
18	unfair	7.88	2.12
18	unfair	7.95	1.05
18	unfair	8.88	1.12
18	unfair	9.18	1.82

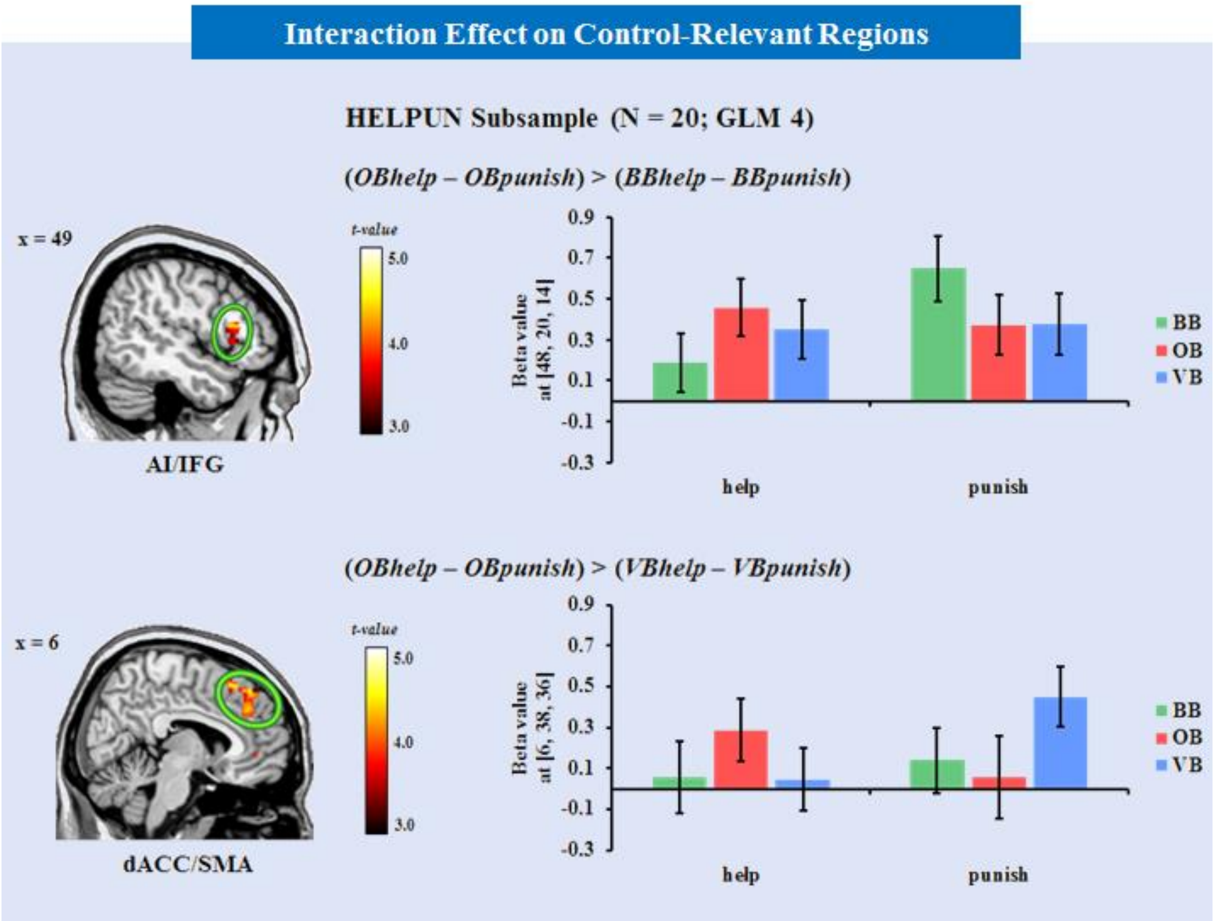

Supplementary Figure S1 The interaction effect between other-regarding attention and altruistic choice on the control-relevant regions based on HELPUN subsample (GLM4). Display threshold: $p < 0.005$ (unc.), $k = 50$. BB = baseline block, OB = offender-focused block, VB = victim-focused block; AI = anterior insula, dACC = dorsal anterior cingulate cortex, IFG = inferior frontal gyrus, SMA = supplementary motor area. The bar plots represent the contrast values of the peak voxels (MNI coordinates in parentheses). Error bars represent the SEM.
